# The deleterious effect of postpartum pyometra on the reproductive indices, the metabolic profile, and oxidant/antioxidant parameters of dairy cows

**DOI:** 10.14202/vetworld.2021.329-338

**Published:** 2021-02-05

**Authors:** Yahia A. Amin, Rana A. Ali, Samer S. Fouad, Rawia M. Ibrahim

**Affiliations:** 1 Department of Theriogenology, Faculty of Veterinary Medicine, Aswan University, Aswan, Egypt; 2 Department of Zoology, Faculty of Science, South Valley University, Qena, Egypt; 3 Department of Clinical Pathology of Veterinary Medicine, Qena University Hospital, South Valley University, Qena, Egypt; 4 Clinical Laboratory Diagnosis, Department of Animal Medicine, Faculty of Veterinary Medicine, South Valley University, Qena 83523, Egypt

**Keywords:** dairy cows, metabolic profile, oxidant/antioxidant parameters, postpartum pyometra, reproductive indices

## Abstract

**Background and Aim:**

Postpartum uterine infectious diseases, such as pyometra, have discrepancy with both health and, subsequently, productivity of dairy cows due to its high prevalence and the high cost of treatment. Therefore, this study investigates the influence of pyometra on the reproductive indices, the metabolic profile, and oxidant/antioxidant parameters of the pyometric animal compared to those of healthy ones.

**Materials and Methods:**

The study included 30 cows. The animals were differentiated into two groups of 15 cows each: A group of pyometra and a control group. All pyometric cows were subjected to breeding soundness examination after the end of pyometra and were compared to the control group. Blood samples were obtained to assess the levels of glucose, non-esterified fatty acids (NEFA), triglycerides (TGs), cholesterol, albumin, total protein, alanine aminotransferase, aspartate aminotransferase (AST), alkaline phosphatase (ALP), blood urea nitrogen (BUN), creatinine, calcium (Ca), phosphorus, sodium, potassium, progesterone hormone (P4), malondialdehyde (MDA), total antioxidant capacity (TAC), glutathione peroxidase (GPx), and superoxide dismutase.

**Results:**

Results revealed significant prolonged duration of first estrus, the days open, and the required number of services due to pyometra. The pyometra group yielded increased levels of NEFA, TGs, ALP, BUN, creatinine, MDA, and progesterone hormone. In addition, significant decrease in the levels of glucose, cholesterol, albumin, Ca, phosphorus, sodium, TAC, GPx, and superoxide dismutase was observed in the pyometra group. Finally, no difference in the concentrations of total protein, ALT, AST, and potassium was observed in the pyometra group.

**Conclusion:**

The reproductive indices was adversely influenced in cows with postpartum pyometra, and metabolic profile, involving energy balance signals and liver function indicators, revealed differences between the two groups. Increased levels of oxidative stress parameters and decrease levels of antioxidant levels were also found, suggesting that pyometra is an incentive for oxidative stress. Overall, checking the energy balance, metabolic imbalances, and oxidant/antioxidant profile, accompanied with pre-emptive procedures during the postpartum period, is essential and can reduce the chances of such diseases and possible noxious results in highly productive cows.

## Introduction

Infection of high-producing cows during the postpartum period is a big contributing factor of different deleterious infectious diseases, such as endometritis, metritis, and pyometra, which greatly affect the health and productivity of dairy cows. Needless to say, such diseases are considered the most problematic diseases due to the high cost of treatment, especially when accompanied by high prevalence among dairy cattle [1].

Pyometra is the exposure of the uterus to infection leading to cumulating of pus inside its lumen while the ovary exhibits presence of a persistent corpus luteum [2]. A closed cervix is common in most cows; however, in few cases, closure of the cervical lumen is not fully completed, and therefore, purulent discharge can be seen come out from the vagina when the cow lies down, urinates, or defecates [3]. Moreover, it was reported that pyometra was considered a branch of endometritis in cases where ovulation occurs in cows suffering from contamination of the uterus [4].

Cows need physiological adaptations during the critical transition period, which requires increase in the energy requirement to meet the milk yield requirement. However, in this period, dairy cows lose appetite for food, which is the reason for a negative energy balance (NEB) [5]. Uterine health problems occurring at postpartum during early lactation are related to a simultaneous occurrence of severe NEB [6]. During invasion of the uterus by the infection, pro-inflammatory cytokines alter the levels of the acute-phase protein in the plasma [7], which adversely influence the uterine immunity [6]. This reveals that the alteration of the immune status is accompanied by NEB in postpartum dairy cows. Therefore, the nutritional status is considered the most critical factor that affects immune cell function [8].

The NEB can be detected by examination of different metabolites such as non-esterified fatty acids (NEFA) and beta-hydroxyl butyrate. Diversity in these metabolites was not evident when measured in the transition period 3 weeks before and after calving in the case of endometritis. Energy balance before calving was similar, yet NEB in the first 3 weeks postpartum was more serious in the case of endometritis than that in the healthy ones [9].

Furthermore, nutritional status is not only critical to immune cell function but also it is also considered a long-run factor affecting reproductive achievement of cows, given the strong connection among resumption of ovarian activity during the postpartum period and energy balance. Butler *et al*. [10] observed that NEB suppressed the production of gonadotropin-releasing hormone from the hypothalamus, subsequently suppressing the production of the luteinizing hormone (LH) from the anterior pituitary, ultimately leading to reduced responsiveness of the ovary to LH. Suppression of production of such hormones impedes development of dominant follicles, which further leads to the demise of ovarian periodic activity.

Diagnosis of uterine health problems depends on the palpation of the uterus and the vaginal exudate [11]. It was reported that vaginal exudate was accompanied with increased oxidative stress [12]; however, the correlation between uterine health status, metabolic profile, and oxidative stress is yet unknown.

The subject of this study is mainly concerned with diseases of the postpartum period (pyometra) due to its great influence on the reproductive life and its effect on the future fertility of the high-producing cows. Adequate knowledge of metabolic status, oxidant/antioxidant profile, and the action of the reproductive organs during the postpartum period could provide important information on the diagnosis, treatment, and prevention protocols for postpartum pyometra. We hypothesized that pyometra causes adverse alteration in the metabolic status and oxidant/antioxidant profile, as well as the liver and kidney functions.

This study aimed to investigate the changes of reproductive indices in pyometric dairy cows compared to those of healthy cows, with special reference to its deleterious effect in the metabolic profile and oxidant/antioxidant parameters which may point to a new direction for the further studies of the pathogenesis, treatment, and prevention of postpartum pyometra.

## Materials and Methods

### Ethical approval

All procedures were performed according to the guide approved by the Ethics Committee of the Faculty of Veterinary Medicine, Aswan University, Egypt.

### Animals

The study included 30 Holstein Friesian cows. The animal subjects belong to a private dairy farm in Qena Province, Egypt. The study was carried out between January and December 2019. The mean parity of the cows was 3-5, aged 5-7 years, weight 250-350 kg and the BCs were 2.8 (scale: 1=thin, 5=fat) [13]. The cows undergo examination every week after calving. A total mixed ration was used for feeding of cows and comprising corn silage, grass silage, and concentrates. The animals were milked twice daily with a milking machine, and their daily milk production ranged from approximately 25 kg of milk per head.

The cows were categorized into two groups of 15 cows each: The pyometra group and the control group. Fifteen pluriparous cows with a history of clinical endometritis were examined and diagnosed with pyometra (group of pyometra, Group A) [14]. The remaining 15 cows, which belong to the control group (Group B), had a history of normal parturition and no postpartum disease; ultrasonographic examination confirmed absence of CL in the ovary, and the uterus is free from accumulated fluids. All pyometric cows were subjected to breeding soundness examination after the treatment of pyometra with administration of PGF2α injection (2 mL of ESTRUMATE, 500 mcg cloprostenol, per cow through intramuscular injection) and were then compared to the control group. The investigation involved the following breeding parameters: Duration to 1^st^ estrus (d); days open (d) (the end of voluntary waiting period which was considered 50 days to successful insemination); and the number of service per conception.

### Blood sampling

Blood samples were collected from the jugular vein of the animals [15]. Samples were taken weekly for 20 weeks once pyometra was confirmed. Blood samples were collected from three types of evacuated tubes: The first one was coated with EDTA for evaluation of the NEFA, the second type was coated with sodium (Na) fluoride for the determination of glucose levels, and the third type was a plain evacuated tube for separation of serum to measure oxidant/antioxidant parameters and other biochemical parameters. Plasma and serum samples were separated and stored at −20°C till further analysis. Samples underwent further analysis for the detection of progesterone concentration, metabolic profile, biochemical reaction, and oxidant/antioxidant parameters.

### Hormonal analysis of progesterone levels (P4)

Serum P4 levels (ng/dl) were measured using radioimmunoassay (RIA) kits according to Kubasik *et al*. [16].

### Determination of energy-related parameters

Serum biochemical parameters were measured using CHEM-7 (a next-generation automatic clinical biochemistry analyzer, ERBA Diagnostics Mannheim GmbH, Mannheim, Germany).

An enzymatic colorimetric method was used to detect glucose using Biodiagnostic kits. NEFA was assessed using NEFA kits (Colorimetric, Randox Reagents, Randox Laboratories Ltd., United Kingdom), according to the manufacturer’s instructions.

Triglyceride (TG) was evaluated using TG GPO-POD Liquid kits (Spinreact). Enzymatic colorimetric method was employed for measuring cholesterol using kits from Biodiagnostic. Albumin was measured through colorimetric method using kits from Biodiagnostic. Finally, total protein was measured through protein biuret method using kits from Biodiagnostic. Alanine aminotransferase (ALT), aspartate aminotransferase (AST), and alkaline phosphatase (ALP) activity were detected by colorimetric method using kits from Biodiagnostic. Blood urea nitrogen (BUN) detection was done through the Berthelot method using kits from Spectrum, while creatinine detection was done through colorimetric method using kits from Biodiagnostic, according to the manufacturer’s instructions.

### Determination of metabolism-related minerals

Calcium (Ca) was detected through Arsenazo III (Spectrum) kits; phosphorus and sodium through colorimetric method using kits from Biodiagnostic; and potassium (K) through turbidimetric method using kits from Biodiagnostic.

### Oxidant/antioxidant parameters

Levels of malondialdehyde (MDA), superoxide dismutase (SOD) activity, and total antioxidant capacity (TAC) were determined using commercial ELISA kits (Cayman, USA), according to the manufacturer’s instructions. Glutathione peroxidase (GPx) was determined through diagnostic kits (Sigma Aldrich USA), according to the manufacturer’s instructions.

### Statistical analysis

Data are described as means±SE. Data analysis was performed using SPSS Statistics 19 software (IBM, USA). Comparison of the groups was done using a t-test. p< 0.05 was considered statistically significant.

## Results

Results revealed presence of purulent discharge flow out from the vagina of cows suffered from pyometra accompanied with uterine distention.

### Adverse effect of pyometra on the fertility profile

Compared with the control group, the affected pyometric group showed a significant increase in the duration to first estrus, the days open, and the required number of services per conception (Table-1). Concerning P4 concentrations, the pyometric cows have significant higher values than the control cows.

**Table-1:** Fertility parameters and progesterone concentration in cows suffered from pyometra compared to healthy ones.

Parameter	Pyometra group (Group A) (n=15)	Control group (Group B) (n=15)
Progesterone (P4) ng/dl.	1.15±1.55*	0.68±1.78
Duration to 1^st^ estrus (d)	91.36±1.25*	35.69±1.56
Days open (d)	120.21±2.01*	45.26±1.58
Number of services (n)	2.25±0.26*	1.30±0.19

Values are expressed as Mean±SE. Asterisks (

* ) indicate statistically significant (p<0.05)

### Energy status and biochemical profile of pyometra

**Figure-1:**
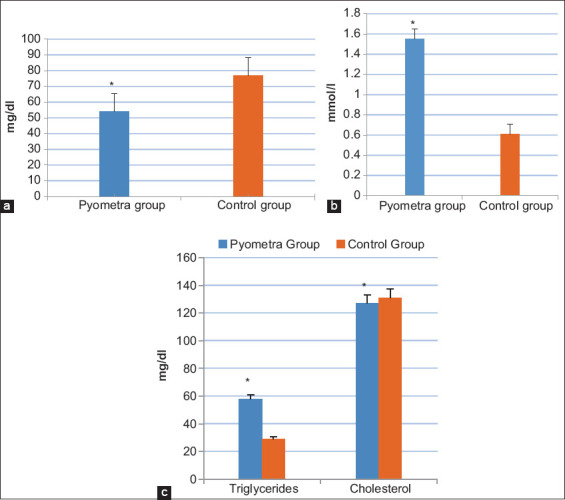
Concentrations of energy-related parameters in cows suffered from pyometra (pyometra group) compared to healthy cows (control group); (a) blood glucose levels (mg/dl), (b) serum non-esterified fatty acids levels (mmol/l), and (c) serum triglycerides and cholesterol levels (mg/dl). Values are mean±SE. Asterisks (*) indicate statistically significant (p<0.05).

**Figure-2:**
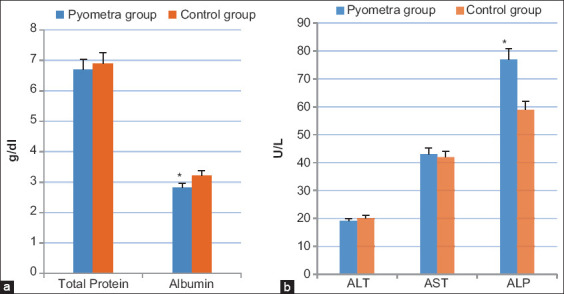
Concentrations of liver function profile in cows suffered from pyometra (pyometra group) compared to healthy cows (control group); (a) serum total protein and albumin levels (g/dl) and (b) serum ALT, AST, and ALP levels (U/L). Values are mean±SE. Asterisks (*) indicate statistically significant (p<0.05).

**Figure-3:**
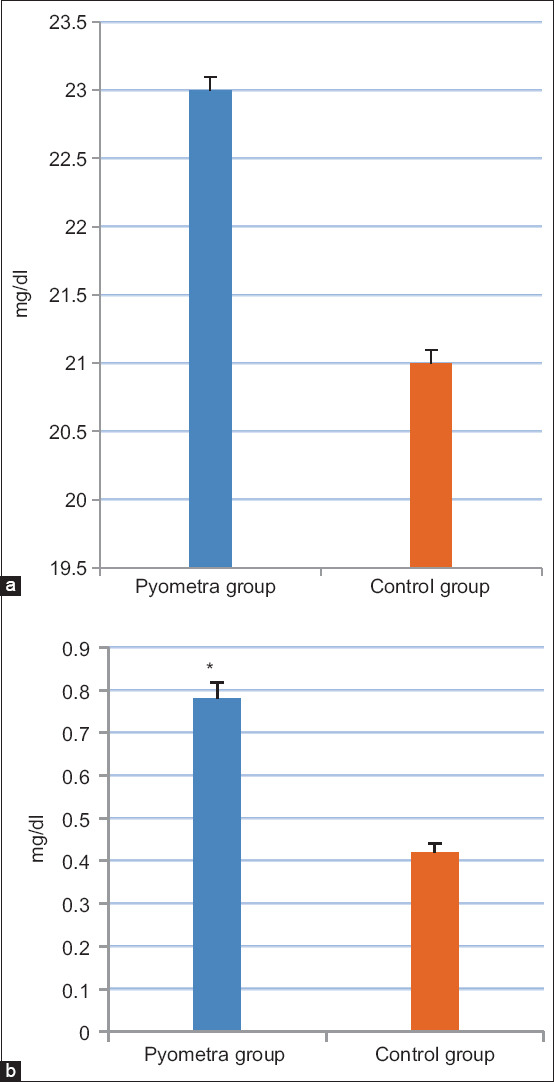
Concentrations of kidney function profile in cows suffered from pyometra (pyometra group) compared to healthy cows (control group); (a) blood urea nitrogen levels (mg/dl) and (b) creatinine levels (mg/dl). Values are mean±SE. Asterisks (*) indicate statistically significant (p<0.05).

Results revealed that blood glucose concentration of pyometric cows was significantly lower than that of the healthy cows (Figure-1a). A significant increase (p<0.05) was observed in the serum NEFA levels and TGs in pyometric cows compared to that in the control group (Figure-1b and c). In contrast, cholesterol and albumin were lower in the pyometric cows compared to the control group (Figures-1 and 2). Moreover, total protein values yielded no significant difference in the pyometric group compared to healthy ones (Figure-2a). The liver function parameters (AST and ALT) were within the normal range while ALP appeared to be increased in the pyometric group (Figure-2b). Finally, a temporary increase of BUN and creatinine was observed in the pyometric group (Figure-3).

### Determination of metabolism-related minerals

**Figure-4:**
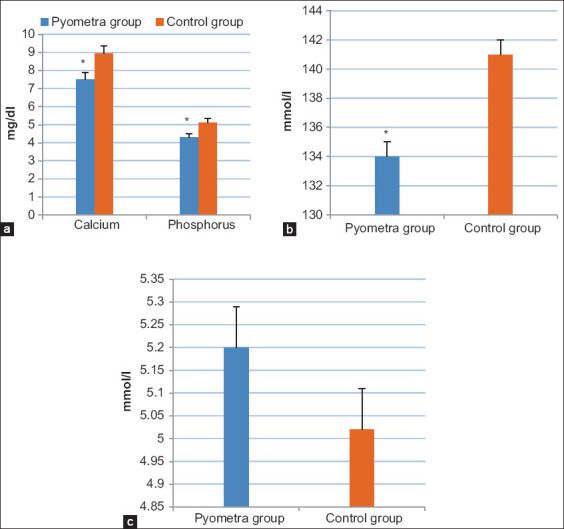
Concentrations of metabolism-related minerals in the group of cows suffered from pyometra compared to the control group; (a) serum calcium and phosphorus levels (mg/dl), (b) serum sodium levels (mmol/l), and (c) serum potassium levels (mmol/l). Values are mean±SE. Asterisks (*) indicate statistically significant (p<0.05).

It was found that Ca, phosphorus, and sodium concentrations in the pyometric group were lower (p<0.05) compared to those in the healthy group (Figure-4). In the contrary, a potassium concentration was notably lower in the pyometric group than the healthy group (Figure-4).

### Oxidative stress and antioxidant profile examination

Pyometra causes significant increase (p<0.05) in MDA levels and significant decrease in the levels of SOD, GP_X_, and TAC (Table-2).

**Table-2:** Oxidative stress and antioxidant profile in cows suffered from pyometra compared to healthy ones.

Parameter	Pyometra group (Group A) (n=15)	Control group (Group B) (n=15)
MDA (µmol/L)	8.60±0.16	3.33±0.06*
Total antioxidant capacity (TAC) mmol/ml	0.32±0.21*	0.65±0.26
Glutathione peroxidase (GPx) U/mL	0.52±0.20*	0.94±0.23
Superoxide dismutase (SOD) U/mL	144±0.19*	153±0.12

Values are expressed as Mean±SE. Asterisks (

* ) indicate statistically significant (p<0.05)

## Discussion

Uterine infection is one of the most serious problems in dairy cows. This study is considered the first to investigate the reproductive indices, the metabolic profile, and oxidative stress biomarkers in the case of cattle pyometra.

The influences of uterine disease on the ovarian and uterine expressions either on the short or long runs are not completely elucidated; however, evidences proved that uterine disease is a factor affecting fertility of dairy cattle. Studies reveal a diminish in reproductive performance as a consequences of prolonged inflammation involving the uterine tube [17,18].

The present study investigated the effect of pyometra on the reproductive indices, the energy status, the associated metabolic changes, and oxidant/antioxidant parameters in crossbreed dual-purpose cows during the postpartum period.

A significant increase was found in the duration to first estrus, the days open, and the required number of services per conception in the affected pyometric cows compared to those of the control cows. This means that pyometra negatively affects the fertility parameters, which suggests a greater need for a better understanding of pyometra associated parameters to minimize the economic losses due to low fertility.

Pyometra is an intrauterine accumulation of pus accompanied by persistent corpus luteum. When luteolysis is prevented in pyometric cows due to failure of the endometrial luteolytic factors or endogenous prostaglandin, the animal is unable to undergo a full cycle. This explains pyometric cows showing increase in both the average days open and the duration to the first estrus in the current study. This is also supported by the values of P4 concentrations in the present study, which were found to be significantly higher in the pyometric cows. Therefore, it was reported that the best treatment of such cases is the administration of prostaglandin F2α or its analogs at normal luteolytic doses. About 80% of the treated cases happened mainly by evacuation of the exudate and bacteriologic clearance of the uterus. Although such treatment causes a decrease in the first-service conception rate, three or four inseminations may be required to successfully conceive most cows, while 20% of the cows needed to repeat treatment. Hence, prostaglandin treatment does not need to be accompanied with intrauterine treatment.

This study describes the presence of the robust relation between infection with pyometra and the energy state during the postpartum period of pyometric cows. The parameters belonging to energy status markedly altered in the pyometric cows. Blood glucose concentration in pyometric cows was significantly lower than that in the healthy cows. In a previous research, it was reported that glucose level was reduced during the partum and postpartum periods of Holstein cows [19], which might be due, in part, to inadequate dry matter intake and increasing demands due to mammary gland development and milk production [20]. These findings are in accordance with those of Nazifi *et al*. [21], in which cows with infected uterine showed disturbances in the energy metabolism till 4 weeks postpartum; furthermore, the physiological adaptations that were needed to meet energy needs were delayed.

Senosy *et al*. [22] observed that the decrease in the glucose level in cows suffering from endometritis at 4 and 6 weeks of postpartum might potentially confirm this delay in the physiological adaptations.

Another explanation for the decrease of glucose depends on the process of gluconeogenesis which occurs in the liver and considered the principal source of blood glucose in ruminants. At calving, healthy cows reveal an increase in the glucose level [23]. The high demand for lactose synthesis a few weeks after calving may be the cause of the decreased levels of glucose at that time [23,24]. In the pyometric group, glucose concentration is lower than the normal range, which may be attributed to the effect of deteriorated liver function on gluconeogenesis [24,25].

In this study, low glucose levels caused energy insufficiency which resulted in some metabolic events ultimately causing increased fat mobilization. Therefore, NEB is responsible for the redundant fat mobilization which leads to the increase in the levels of NEFA [26]. Results in this study also revealed a significant increase in the NEFA levels in the pyometra group. It was reported that the adaptation of cows to NEB can be measured by evaluation of the glucose concentrations in association with NEFA concentrations in the blood of such cows [27]. The mobilized NEFA represents the alternative energy source which plays an important role in preserving the limited available glucose [28]. Mili *et al*. [29] observed an increase in the NEFA concentration besides the decrease in the glucose and Ca concentrations predispose to the increased risk of postpartum uterine infections. It is also worth noting that the increase in the NEFA levels during the transition period [30] was associated with the development of clinical postpartum disorders such as puerperal metritis in cows [31], ketosis [30], and retained fetal membranes [32]. However, these findings do not agree with those reported by Ghanem *et al*. [33], in which total NEFA concentrations in cows with persistent bacterial infection did not differ from those in cows without infection.

In the current study, TG levels in the pyometra group were significantly higher than those in the control group; this suggests high-fat mobilization in pyometric cows. Previously, it was confirmed that cows suffering from metritis exhibited higher levels of TG due to its more energy needs [34].

Moreover, in the present study, there is a decrease in the cholesterol level in pyometric cows. Detection of cholesterol levels in the serum of dairy cows, especially during the postpartum period, was used as an indicator of disease risk [35]. Semacan *et al*. [36] concluded that there was a lowering in the concentrations of cholesterol in cows with clinical disorders compared to healthy cows. The reason for decreasing levels of cholesterol in pyometra group may include the decreased in feed intake, especially the origin of the cholesterol is mostly from the intestine of the cows [37]. All these findings strongly proved that metabolic imbalance and NEB predispose the increased risk of pyometra and vice versa.

In addition, the study also observed a reduction in the serum albumin levels in the pyometric cows, while no significant difference was found in total protein values. These findings are almost similar to those reported by Shah *et al*. [38], wherein hyperproteinemia and hypoalbuminemia were found in pyometric female dogs and the increased levels of total protein were attributed to the acute-phase reaction in pyometric dogs [39], while the hypoalbuminemia resulted from the renal loss of albumin.

Results in this study are also consistent with those found by Ramadan *et al*. [31], wherein a lower level of albumin was detected during the parturition and the postpartum periods, but with no much variation in total protein.

AST and ALT are extremely useful and are considered specific indicators of hepatocyte injuries [40,41]. In our study, the levels of liver function enzymes were observed in normal range, except for ALP which was increased in pyometric cows during the postpartum period. The same finding was found by Shah *et al*. [38] as ALP was increased in pyometric dogs, whereas AST and ALT were within the normal range. Similarly, a study conducted on pyometric mares revealed that all mares affected with pyometra exhibited increased levels of AST and ALP when compared with control mares [42]. Moreover, this rise in the levels of ALP has been theorized to be a result of intrahepatic cholestasis [43].

In another study on the liver function parameters and TG in cows suffering from metritis, results indicate that the levels increased in the metritis group compared to those in the control group [44].

A kidney’s state is evaluated through BUN and creatinine. Several factors affect the BUN, such as renal excretion and urea recycling in rumen [40,45]. Creatinine symbolizes skeletal muscle mobilization and could be altered through the kidney without reabsorption [46].

In the current study, evaluation of BUN and creatinine revealed presence of a tentative rise on the pyometra group, which may initiate muscle tissue mobilization resulting from the stress of birth [47]. Few studies investigated the level of BUN in pyometric cows. One study carried out by Shah *et al*. [38] who showed that values of BUN and creatinine were higher in pyometric dogs compared to the healthy ones. According to their study, the increased values of BUN indicate that the efficiency of kidneys to remove nitrogenous waste from the circulation is affected [48]. In contrast, Ghanem *et al*. [33] declared that cases of persistent bacterial infection have decreased levels of BUN compared with healthy cows.

In a study by Cui *et al*. [44], no obvious difference was noticed in BUN between cows suffering from metritis and cows that are healthy. Furthermore, in cows with endometritis, Giuliodori *et al*. [49] reported no change of BUN. Ramadan *et al*. [31] gone further to illustrate that levels of urea were significantly increased in cows suffering from postpartum metritis, which is a conceivable outcome of increased mobilization of protein and BCS loss.

Regarding mineral profile in the current study, it was observed that the Ca and phosphorus levels in the pyometric group were obviously lower than those in the control group.

Once parturition occurs and lactation begins, a great need for mechanisms of Ca homeostasis at calving arises; therefore, most cows suffer from Ca deficiency at calving [24]. The reduction in levels of such minerals during and after calving corresponded with the findings in our present study. These results are also in accordance with the previous reports [50,51]. Hypocalcemia usually occurs in cows at calving which may result in unfortunate conditions: the first is the higher plasma cortisol concentrations resulting in exacerbation of the immunosuppression status [52] and the second is the reduced muscle tone of the uterus [53]. The possible consequence of these conditions is the increased incidence of postpartum uterine infection.

In the current study, sodium levels were found to be lower in pyometric cows compared to those in the control ones, but in contrary, K levels were higher in the pyometra group. Similarly, Ramadan *et al*. [31] found lower Na levels and higher K levels in metritis cows compared to control subjects. In their study, the decrease in Na levels correlated with great amount of fluid loss during birth, in addition to the production of colostrum. However, in contrary, another study revealed that there were no marked differences in the levels of such minerals at both pre- and postpartum periods [54].

It has been revealed that oxidative stress is accompanied with infectious conditions in various domestic animals [55]. The high reactive oxygen species (ROS) level in animals makes them susceptible to infection due to poor immunity. These ROS cause damage to epithelial cell barriers resulting in tissue injury, thereby making them susceptible to pathogen invasion. Lately, it was reported that there is a relationship between oxidative stress and pyometra from bacterial infection [42].

The antioxidant system suffers from exhaustion when there is a reduction in the antioxidant levels, which leads to a final state of oxidative stress as this system is the only system responsible for the counteract of ROS. In the current study, checking of the levels of MDA revealed that there is a considerable rise in its levels in the pyometric group compared to the healthy group. Moreover, investigation of the antioxidant profile exhibited presence of marked reduction in the levels of different antioxidant components such as SOD, GP_X_, and TAC in the pyometric group compared to healthy control animals.

Thus, the considerable increase in the MDA in the pyometra group indicates the occurrence of lipid peroxidation. Lipid peroxidation is generated from the release of oxygen free radicals [56] as a consequence of bacterial infection (sepsis). Superoxide radical is the greatest harmful one due to its potent suppressor effect on the NO molecule; in addition, its reaction with this molecule creates peroxynitrite which is a serious reactive oxygen species [57]. Therefore, if the endogenous antioxidants (SOD and GSH) failed to remove these radicals, the proportion of oxidation transcends the proportion of antioxidation, and subsequently, oxidative stress will be the final state [58].

The association of an increase in the levels of lipid peroxidation with the decreased in the level of antioxidants has been observed in different animals suffering from pyometra, such as the mare [42], rabbit doe [59], and female dog [60].

One of the important lines of defense against free radicals originated from an endogenous antioxidant enzyme called SOD. An increase in SOD provides protection against toxic oxygen radicals during oxidative stress. Its mechanism of action depends on the scavenging of both intra- and extracellular superoxide radicals to produce hydrogen peroxide and oxygen [61]. This means that SOD has the ability to transform superoxide anion to hydrogen peroxide and subsequent catalysis by catalase, and GPx transforms hydrogen peroxide into water [62]. Therefore, SOD is considered the greatest essential antioxidant enzymes in biological life.

Results in this study are consistent with those observed in the previous studies noting a decrease in SOD in uterine infection cases [42,63].

Similarly, the previous studies on female dogs and mares revealed that SOD levels were lower in the groups of pyometra compared to healthy ones [42,60,64,65], which illustrated that the antioxidant system was impaired. This refers to the dwindling of antioxidant enzymes to frustrate the oxidative stress produced by ROS.

## Conclusion

The reproductive indices was adversely influenced in cows with postpartum pyometra, and the metabolic profile, involving energy balance signals and liver function enzymes, revealed differences between the two groups of cows. An increase in the levels of oxidative stress parameters and a decrease in antioxidant levels were also found. This proved that pyometra is an incentive for oxidative stress. Therefore, checking the energy balance, metabolic imbalances, and oxidant/antioxidant profile accompanied with pre-emptive procedures during the postpartum period are highly recommended while it helps minimize if not prevent occurrence of such diseases and possible noxious results in highly productive cows. This study is a step to expand the field of future research in the pathogenesis and prevention of pyometra in cows. Further studies are required which should aim at investigating the changes occurring in cows suffering from pyometra from the prepartal to the postpartum period.

## Authors’ Contributions

YAA conceived and designed the study. YAA and RAA executed the experiment. YAA and SSF analyzed the samples. YAA and RMI analyzed the data. YAA drafted the manuscript. All the authors interpreted the data, revised the manuscript for necessary intellectual contents, and approved the final manuscript.
